# Increase of Dengue in Pediatric Travelers in Madrid: A Multicentric Retrospective Experience

**DOI:** 10.3390/tropicalmed10090243

**Published:** 2025-08-28

**Authors:** Isabel Mellado-Sola, Sonia Milkova Ivanova, Milagros García López Hortelano, Paula Rodríguez-Molino, Cinta Moraleda, Sara Otero Alambillaga, Rut Fernández Martín, Francisco José Collado Díaz, Aida Sánchez García, Inés Ojeda Velázquez, Begoña Santiago-García, Talía Sainz

**Affiliations:** 1 Pediatrics, Infectious Diseases and Tropical Medicine, University Hospital La Paz, 28046 Madrid, Spain; isabel.mellado@salud.madrid.org (I.M.-S.); mghortelano@salud.madrid.org (M.G.L.H.); paularmolino@gmail.com (P.R.-M.); talia.sainz@salud.madrid.org (T.S.); 2 Pediatrics Department, University Hospital El Escorial, El Escorial, 28200 Madrid, Spain; 3 La Paz Research Institute (IdiPAZ), 28046 Madrid, Spain; 4 Faculty of Medicine, Autonomous University of Madrid (UAM), 28049 Madrid, Spain; 5 Centro de Investigación Biomédica en Red en Enfermedades Infecciosas (CIBERINFEC), 28029 Madrid, Spain; bsantiagogarcia@gmail.com; 6 Pediatrics Infectious Diseases, University Hospital 12 de Octubre, 28041 Madrid, Spain; cintamoraledaredecilla@gmail.com (C.M.); soteroalambillaga@gmail.com (S.O.A.); rut.fernandez98@gmail.com (R.F.M.); 7 Instituto de Investigación Sanitaria Hospital 12 de Octubre (imas12), Fundación Biomédica del Hospital, Universitario 12 de Octubre (FIB-H12O), 28041 Madrid, Spain; 8 Clinical Analysis Department, University Hospital Niño Jesús, 28009 Madrid, Spain; franciscojose.collado@salud.madrid.org; 9 Microbiology Department, University Hospital Niño Jesús, 28009 Madrid, Spain; aida.sanchez.garcia@salud.madrid.org; 10 Pediatrics and Infectious Diseases, University Hospital Gregorio Marañón, 28007 Madrid, Spain; ines.ojeda.velazquez@gmail.com; 11 Instituto de Investigación Sanitaria Gregorio Marañón, 28009 Madrid, Spain

**Keywords:** dengue, children, international travelers, VFR (visiting friends and relatives)

## Abstract

Dengue fever has significantly increased globally, extending into non-endemic regions. This study aims to describe the epidemiological and clinical characteristics of pediatric dengue cases diagnosed in Madrid, Spain, over ten years. We conducted a retrospective observational study across four tertiary hospitals, including all confirmed dengue cases in children under 16 between 2015 and 2024. Epidemiological data, clinical presentation, laboratory findings, and outcomes were collected, with severity assessed according to the 2009 WHO criteria. Forty-six cases were identified, with 72% diagnosed in the last three years and a peak incidence in 2024. Children visiting friends and relatives (VFR) constituted the majority of cases (56%). The most frequent clinical features were fever (100%) and gastrointestinal symptoms (78%), while laboratory findings included leukopenia (72%), thrombocytopenia (70%), and hypertransaminasemia (74%). Five cases (10%) met the criteria for severe dengue, one being fatal in a patient with pre-existing oncological disease. We identified no autochthonous cases. These results highlight the growing impact of imported pediatric dengue in non-endemic regions, the particular vulnerability of VFR travelers, and the need for clinical awareness, improved diagnostic availability and prevention strategies, especially in climate-influenced vector expansion.

## 1. Introduction

Dengue fever is caused by one of the four serotypes of dengue virus (DENV), transmitted by *Aedes* mosquitoes (*Aedes aegypti* and *Aedes albopictus)* [1]. In recent years, dengue cases have risen in tropical and subtropical areas in Asia, Africa, and the Americas, posing a growing global health challenge [1,2,3]. Cases peaked in 2024, with over 13 million cases reported, making it the highest year on record. Acute cases have been reported in travelers, both children and adults, coming from endemic to non-endemic areas, with numbers rising significantly in the last 2–3 years [4]. As dengue vectors are present in regions where dengue is not endemic, imported cases may pose a public health risk. Autochthonous dengue cases have been reported in some European countries in 2023 and 2024 (Italy 295, France 128, Spain 11), and surveillance programs are ongoing [4,5,6]. Unfortunately, data are not always disaggregated by age, and specific pediatric data are lacking. The most recent analysis from the international network GeoSentinel, which examined travel-associated dengue from 2007 to 2022, describes an increase in the global number of symptomatic dengue cases and dengue-related deaths between 1990 and 2019; additionally, in 2022, a substantial number of dengue cases were imported into Europe [7].

We describe the epidemiological and clinical characteristics of dengue cases diagnosed in children in four tertiary hospitals in Madrid, Spain, in the last ten years.

## 2. Materials and Methods

We conducted a retrospective observational study that included data from four tertiary hospitals in Madrid, Spain. All cases of children (under 16 years of age) diagnosed with dengue from January 2015 to December 2024 were reviewed. The inclusion criteria were as follows: age < 16 years and confirmed dengue fever (see laboratory diagnosis below). Cases were diagnosed according to routine clinical practice, with no unified protocol across centers, which may have different diagnostic tests available for diagnosis. Epidemiological and clinical data, including clinical presentation, diagnostic tests, coinfections, and treatment-related information, were electronically collected and stored in a standardized, anonymized database. The definition for VFR children includes both children born in endemic regions who later immigrated to Spain and returned to visit family, and children born in Spain to immigrant parents. Follow-up data included symptom duration, intensive care admission, blood tests, and laboratory diagnosis, including repeated serologies where available, until discharge. We assessed clinical and laboratory severity criteria using the 2009 WHO clinical guidelines [1]. Dengue was considered severe if there was shock, plasma leakage, respiratory compromise, severe bleeding, or severe organ damage.

Direct and indirect methods were used for laboratory diagnosis, and both confirmed and probable cases were included. The definition for confirmed cases was based on at least one of the following diagnostic tests: positive dengue non-structural protein 1 antigen of dengue (NS1) or positive Polymerase Chain Reaction (PCR) for viral RNA. The presence of dengue IgM antibodies in a serum sample was considered a probable dengue diagnosis. Primary dengue was defined as laboratory-confirmed dengue with one of the following criteria: (a) positive NS1 or IgM with recent travel preceding the infection being the first ever to a DENV endemic region, or (b) positive NS1 or IgM in the absence of IgG. Probable secondary dengue was defined as a DENV infection either documented in a prior episode, supported by a medical report of previous dengue, or indicated by compatible serology (IgM−/IgG+). Quantitative antibody titers were not available to assess for a significant rise, which limited our ability to distinguish between primary and secondary dengue infection. The dengue RDT used was the immunochromatographic Biosynex^®^Dengue NS1 assay (Biosynex SA, Illkirch-Graffenstaden, France); the PCR used was the LightMix^®^ Modular Dengue Virus Assay (Roche, Madrid, Spain); for serology, the chemiluminescence test VIRCLIA IgM/IgG mono test (Vircell, Santa Fe, Granada, Spain) and an enzyme-linked immunosorbent assay ELISA IgM/IgG Capture^®^DX select kit (Diasorin, Alcobendas, Madrid, Spain) were used.

Definitions for hematological variables were as follows: leukopenia < 4500 leukocyte/mcL, leukocytosis > 10,000–12,000 leukocyte/mcL, lymphopenia < 1500 lymphocytes/mcL, and thrombocytopenia < 100,000 platelets/mcL. Anemia varied depending on age (<2 years < 10.5 g/dL; 2–12 years < 11.5 g/dL; >12 years < 12 g/dL for females and <13 g/dL for males) [8]. Hypertransaminasemia was considered if ALT > 35 U/L or AST > 40 U/L, and hyperbilirubinemia if the total bilirubin was > 1 mg/dL [9].

The study received ethical approval from the Hospital’s Ethics Committee (PI-1370). No informed consent/assent was required due to the retrospective design of the study (PI-1370).

Continuous variables were described using medians and their interquartile ranges (IQRs), whereas categorical variables were described using absolute and relative frequencies. Figures were generated using Microsoft Excel. All analyses were performed using STATA v18, SPSS (version 25), and/or Microsoft Excel.

## 3. Results

**Figure 1:**
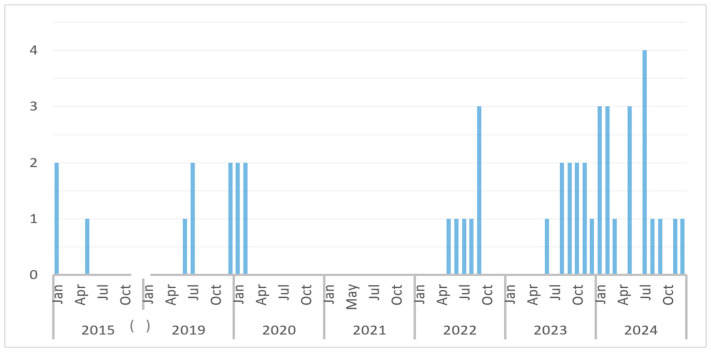
Distribution of dengue cases during the study period. The total number of cases each month is represented by bars. (The brackets highlight the gap between 2015 and 2019, from which we have no cases).

Forty-six cases diagnosed with dengue were included in the study, with 76% of cases (35/46) diagnosed in the last three years, showing an increase in the incidence, mostly in 2024 (Figure 1).

The median age at diagnosis was 10 years (IQR 6–14). Forty percent (18/46) were female, and 56% (26/46) of cases corresponded to children visiting friends and relatives (VFR), of which 65.4% (17/26) were children born in Spain to immigrant parents. Another 24% (11/46) were children residing in endemic countries who had just arrived, and the remaining 15% (7/46) were travelers for tourism born in Spain. The most frequent travel destination was the Dominican Republic (10/46, 22%), followed by Cuba (7/46, 15%), Paraguay, and Colombia (4/46, 8.6% each). Other destinations included Central and South American countries, Southeast and Central Asia (7/46, 15%), and Equatorial Guinea (1/46, 2%). The median travel time was 30 days (RIC 20–60), obtained in 23/46 patients (50%). Most cases were diagnosed in the summer (15/46, 33%), during school holidays, or in January, following the Christmas break (8/46, 17%). None of the patients had received any dengue vaccine before the dengue episode.

The main reason for consultation in the Emergency Department (ED) was fever following international travel; all cases (46/46, 100%) presented with fever (temperature > 38 °C). Gastrointestinal symptoms were more frequently present than rash. Eight patients presented with bleeding, three of whom had hematemesis; the rest presented with mild oral mucosal bleeding. Two patients presented with sleepiness (See Table 1). No other warning signs for severe dengue were present in any of the cases in the ED (severe abdominal pain, persistent vomiting, rapid breathing, restlessness, blood in stool, being very thirsty, or pale and cold skin).

The managing pediatricians performed blood tests at the ED in 45 out of 46 patients (97%). Leukopenia was present in 73% of cases (33/45), lymphopenia in 55% (25/45), and thrombocytopenia in 71% (32/45). Hypertransaminasemia was present in 75% of patients (34/45), but no patient presented hyperbilirubinemia.

All cases met the criteria for confirmed or probable dengue. The two cases classified as probable did not have NS1 or PCR performed, but had a positive IgM. There was no overlap between NS1 and PCR-positive cases. A previous dengue infection was self-reported in 5/46 (11%). Although not medically documented, prior infection was inferred as a secondary dengue infection based on current serologic profiles in the other 5/46. Information regarding serotype was not available as it is not performed routinely. Details regarding diagnostic testing are shown in Table 2.

In total, 29 patients were admitted to the hospital (63%, 29/46) during the study period, and the median length of hospital stay was 3.5 days (IQR 2–5).

Five cases met the criteria for severe dengue during the first 24 h of admission, one in 2023 and four in 2024, with a median age of 11 years (IQR 6–12). Detailed clinical data can be found in Table 3.

The severe case in 2023 had relapsing leukemia, presented with fever and bleeding (hematemesis, epistaxis, and haematoma), and developed septic shock during the first days after admission, and unfortunately died, probably related to the oncologic condition. Regarding the remaining four cases from 2024, only one presented comorbidity (obesity). Two of the 2024 cases required Pediatric Intensive Care Unit (PICU) admission due to hypotension and capillary leak, and required fluid expansion and vasoactive drugs; none required platelet transfusion. The other two severe dengue patients had gastrointestinal bleeding, mild ascites, and a tendency to sleepiness during the first hours of admission, and one of them presented with hypertransaminasemia. Due to the resolution of the symptoms within the first day of admission, they were not admitted to the PICU. Fever and abdominal pain were the most common symptoms at presentation in severe cases in 2024, and hepatomegaly was present in 3/4 at the initial examination. All had thrombocytopenia with a median platelet count of 56,000/mm^3^ (IQR 15,500−106,500). Lymphopenia and hypertransaminasemia were present in 3/4 of cases. One presented an elevation of acute phase reactants suggestive of bacterial coinfection (C-reactive protein 220 mg/L and procalcitonin of 46 ng/mL). In all 2024 severe cases, the dengue antigen was positive; only one (1/4) patient reported previous dengue infection. No sequelae were reported.

## 4. Discussion

In this series, including pediatric dengue cases in Madrid, Spain, a progressive increase in number is reported, with a peak incidence in 2024, when 80% of the severe cases presented. Children VFR were overrepresented compared to other travelers, and no autochthonous cases were diagnosed. There was only one fatal case that presented critical underlying conditions. Diagnosis was varied, and some diagnostic techniques were not always available. These data underline the need to increase awareness among physicians in non-endemic areas and to generalize access to diagnostic tests.

The tendency observed in this series is consistent with global reports and data from the regions where dengue fever is endemic. Dengue cases in 2024 exceeded the highest number ever recorded in a single year, showing a 230% increase compared to the same period in 2023 [10,11]. In South and Central America, endemic dengue cases were 437% higher than the previous five-year average, with more than 12.6 million cases [12], although severe dengue accounted for less than 0.2% [13]. While most international travelers arriving in Spain come from other European countries, national data show that, despite being fewer in number, travelers from Latin America account for a high proportion of imported dengue cases. This is consistent with immigration statistics, which indicate that the majority of foreigners with residence permits originate from countries such as Ecuador, Peru, and Bolivia. This highlights the importance of considering both travel volume and epidemiological risk when designing public health policies [14,15].

In Spain, the main vacation periods occur during Christmas and the summer. The Christmas holiday typically begins around 22 December and extends through 6 January, encompassing both Christmas Day and Epiphany (Día de Reyes), whereas the summer vacation, particularly in the education sector, spans from late June to early September. This period coincides with the hottest months of the year and is commonly used for travel and family holidays. We could appreciate a higher incidence during and after these vacation periods, when children can travel.

The combination of fever and gastrointestinal symptoms was the main clinical presentation in our series. Fever is the most frequent symptom in all series of children, but the presence of gastrointestinal symptoms can vary (10–40% for abdominal pain and diarrhea, 35–70% for vomiting). Hepatomegaly was more common than splenomegaly, as usually reported. Rash and general symptoms were present only in half of the cases in our series, which aligns with previous cases in children [16,17,18]. As in many other conditions, clinical presentation in children differs from that in adults, leading to delays in diagnosis if clinicians are not aware of pediatric particularities.

On the other hand, general symptoms such as headache, retro-orbital pain, dizziness, and chills are usually more prevalent in adults [16]. A series of over 2000 patients with confirmed dengue from Thailand showed significantly more frequent rash, convulsions, diarrhea, splenomegaly, and hepatomegaly in children under 2 years of age compared with those aged 2–12 [18]. Other reported cases showed that younger children were at a higher risk of hemorrhage than older children, with the odds reducing by 8% for every year of increase in age [18]. We could not verify this in our series, with a median age of patients with bleeding of 9 years (IQR 7−13).

Leukopenia and thrombocytopenia were the two most frequent hematological alterations, as described by other authors, with thrombocytopenia having been associated with progression to severe disease [16,17]. High levels of AST and ALT and low serum albumin during the febrile phase have been associated with progression to severe disease. Moreover, hypertransaminasemia (especially high ALT), bilirubin > 1.2 mg/dL, high lactate levels, and fibrinogen ≥ 400 mg/dL have been proven as predictors of mortality [19,20,21]. Unfortunately, data regarding lactate, fibrinogen, and serum albumin were unavailable for our analysis. Although secondary dengue infection was documented by a medical report in only one of our patients, serological profiles suggestive of secondary infection were identified in some of our patients. Secondary dengue has been significantly associated with progression to severe dengue and mortality in children [16,19,20,21], together with multiple vasoactive drugs and positive fluid balance, which are also known predictors of mortality in severe dengue infection in children admitted to the PICU [20]. Therefore, the identification of secondary infection patterns in our patients may hold prognostic relevance. Fortunately, in our series, only two patients needed PICU admission, requiring fluid expansion and vasoactive drugs, but no mechanical ventilation.

Five cases met the criteria to be considered severe, representing 10% of the series, which is a high percentage compared to the proportion described in the literature [10,13]. The alarm signs we found were severe gastrointestinal bleeding in the form of haematemesis, mucosal bleeding, and a tendency to somnolence, which resolved in the first hours of admission. However, one of the five patients who developed severe dengue had no warning signs. The positive predictive value of the individual warning signs for severe dengue has been previously reported to vary between 12% and 58%, with certain warning signs having higher predictive values than others [22]. One patient had an underlying oncological condition that likely contributed to the fatal outcome. The only other comorbidity present in severe cases was obesity. While it has also been reported to increase the risk of severe dengue fever in adults by 50% [23], few studies have reported data in children [24]. Sangkaew et al. found in their meta-analysis a higher risk of progression to severe dengue in women than in men, but the subgroup analysis failed to prove it in children [19]. Nor have significant gender and age differences in mortality been demonstrated in patients with severe dengue or warning signs [21].

Our data suggest that, together with children migrating from endemic regions, children born to migrant parents visiting their home countries represent the highest-risk group among travelers, while comparative data on the proportion of such populations traveling to endemic countries out of all travelers is not known. Evidence indicates that longer stays in endemic regions correlate with increased risk of dengue infection. The median travel duration for our cohort was 30 days, reflecting prolonged exposure to endemic areas [7]. On top of that, they have a lower rate of pre-travel consultation and a lower risk awareness [25,26,27], most probably because of the health inequalities and sociocultural and economic challenges that this population faces. Recent studies in adults have shown an increase in dengue cases in VFR immigrants, in probable connection with the outbreaks in Latin America and the Caribbean [28]. When traveling, specific interventions are needed to promote adherence to pre-travel consultation with specialized professionals among VFR travelers, both children and their families, four to six weeks before traveling. During these consultations, travelers should be advised about preventing mosquito bites, such as using mosquito nets and repellents, and eliminating permanent water deposits to avoid the spread of vectors. Immunization should also be considered, especially for those at high risk in this population, as there are now options accepted by the regulatory agencies to protect children over 6 years of age [29]. Children with a history of dengue fever should be prioritized as well. Recommendations for post-travel consultation should be provided, as well as information on the management of fever. In the emergency room, we should give special attention to VFR children presenting with fever and gastrointestinal symptoms in the first 15 days after returning from a dengue-endemic area [7]. Pediatricians need to be aware of warning signs to identify cases at an early stage.

Interestingly, during these ten years, we did not diagnose autochthonous dengue cases, as all patients were recent travelers to endemic areas with a high incidence of outbreaks. The months following an important holiday (summer and winter break) had the highest number of cases. Nevertheless, as both *Aedes* mosquitoes and imported dengue viruses are present in our region, we should not overlook autochthonous transmission, especially when the weather conditions in Europe are milder and optimal for mosquitoes, and most travels to endemic areas take place. Autochthonous cases in Spain have been reported mainly in the Mediterranean basin in adult patients who denied having traveled outside the country [5]. As shown in several recent studies [11,30], an increase in temperature due to global warming favors a suitable environment for the spread of emergent disease vectors. Consequently, disseminating the dengue virus could pose a significant risk in non-endemic areas.

From a public health perspective, our findings highlight the urgent need to strengthen preventive strategies for VFR families. This should include increasing access to pre-travel health consultations, promoting targeted educational campaigns about disease risks and symptoms, and ensuring early diagnosis and treatment. Improving prevention before, during, and after travel can significantly reduce the likelihood of introducing imported infections into non-endemic areas. Therefore, infection surveillance strategies should be prioritized, campaigns to raise awareness among healthcare workers in primary and secondary care settings should be promoted, and diagnostic tests should be made available for suspicious cases.

The study’s main limitations include its retrospective design and the small sample size despite its multi-center design. Cases were not managed according to a unified protocol, and not all patients were screened for other imported diseases, such as Zika or the Chikungunya virus, which can lead to cross-reaction. Despite these limitations, this study represents one of the most extensive series of pediatric patients in a non-endemic area and offers valuable data that can inform policymakers and health authorities.

The burden of disease related to dengue fever is significantly increasing in non-endemic regions. Given the epidemiological situation in endemic areas and the increase in people’s movements, the numbers may continue to rise in the years to come. We strongly advocate for a sensitization campaign, focusing preventive efforts on the vulnerable population of travelers VFR, including implementing immunization programs. Clinicians should be aware and able to recognize symptoms, and diagnostic tests for the early phase, such as PCR and NS1 antigen, should be available to avoid underdiagnosis.

## Figures and Tables

**Table 1:** Patients’ symptoms and signs upon arrival to the Emergency Department.

Symptoms n (%)	Total (n = 46)
Fever (>38 °C)	46 (100)
Gastrointestinal	36 (78)
Abdominal pain	22 (48)
Vomiting	23 (50)
Headache	19 (41)
Fatigue	11 (24)
Arthralgia	6 (13)
Myalgia	11 (24)
Mucosal bleeding	7 (15)
Rash	22 (48)
Hepatomegaly	8 (17)
Splenomegaly	5 (11)
Petechiae	7 (15)
Sleepiness	2 (4)
Edema	1 (2)

**Table 2:** Laboratory dengue tests for diagnosis. NS1: Non-structural protein 1 antigen of dengue; PCR = Polymerase Chain Reaction; IgM = Immunoglobulin M; IgG = Immunoglobulin G.

Diagnostic Test, n (%)	Performed	Positive
NS1	40	32 (80)
PCR	4	4 (100)
IgM	40	22 (55)
IgG	40	17 (42)

**Table 3:** Detailed clinical data of severe dengue cases.

	1	2	3	4	5
Year of presentation	2023	2024	2024	2024	2024
Age (y)	5	11	12	7	12
Gender	F	M	F	F	M
Type of traveler	Resident	VFR	VFR born in Spain	Resident	VFR
Risk factors	Leukemia	Obesity	-	-	-
Country of origin	Philippines	Dominican Republic	Peru	Philippines	Cuba
Diagnostic test	IgM+/IgG+	IgM+/IgG−	IgM−/IgG−	IgM−/IgG+	IgM+/IgG+
	NS1−	NS1+	NS1+	NS1+	NS1+
Disease classification	Secondary dengue (probable)	Primary dengue	Primary dengue	Secondary dengue	Secondary dengue (probable)
Clinical manifestations of severe dengue	Fever, hematemesis, epistaxis, haematoma	Fever, abdominal pain, vomiting, headache	Fever, abdominal pain	Fever, abdominal pain, vomiting, headache, hematemesis, sleepiness	Fever, abdominal pain, vomiting, hematemesis, sleepiness
Physical examination	Hepatomegaly, splenomegaly	Rash, hepatomegaly, splenomegaly, hypotension, oedema in lower limbs	Rash, hypotension	Hepatomegaly, ascites, pulmonary edema	Hepatomegaly, splenomegaly, ascites
White blood cell	409,600/mcl	19,580/mcl	5870/mcl	6830/mcl	2230/mcl
Lowest lymphocyte count	38,060/mcl	1680/mcl	350/mcl	590/mcl	740/mcl
Lowest platelet count	17,000/mcl	96,000/mcl	117,000/mcl	16,000/mcl	15,000/mcl
Highest transaminase value	AST 51 UI/L	AST 1063 UI/L	AST 20 UI/L	AST 161 UI/L	AST 705 UI/L
	ALT 21 UI/L	ALT 677 UI/L	ALT 16 UI/L	ALT 76 UI/L	ALT 296 UI/L
Other	Fatal case	Elevated CRP (220 mg/L) and procalcitonin (46 ng/mL)	Low prothrombin activity (56%)		Low prothrombin activity (65%)
Required PICU	Yes	Yes	Yes	No	No

## Data Availability

The data presented in this study are available upon request from the corresponding author due to privacy.
